# Flowering Locus T chimeric protein induces floral precocity in edible citrus

**DOI:** 10.1111/pbi.13463

**Published:** 2020-09-16

**Authors:** Judith P. Sinn, Jeremy B. Held, Chad Vosburg, Sara M. Klee, Vladimir Orbovic, Earl L. Taylor, Tim R. Gottwald, Ed Stover, Gloria A. Moore, Timothy W. McNellis

**Affiliations:** ^1^ Department of Plant Pathology and Environmental Microbiology The Pennsylvania State University University Park PA USA; ^2^ The Huck Institutes of the Life Sciences The Pennsylvania State University University Park PA USA; ^3^ Citrus Research and Education Center University of Florida Lake Alfred FL USA; ^4^ United States Horticultural Research Laboratory United States Department of Agriculture Agricultural Research Service Fort Pierce FL USA; ^5^ Institute of Food and Agricultural Sciences Department of Horticultural Sciences University of Florida Gainesville FL USA; ^6^ Present address: Department of Microbiology University of Washington Seattle WA USA

**Keywords:** grapefruit, blooming, juvenility, early flowering, accelerated breeding

Citrus (family Rutaceae) is one of the most important fruit crops, with world production exceeding 150 million metric tons in 2018 and an international gross production value of 37.5 billion US dollars in 2016 (http://www.fao.org/faostat/en/#data). Citrus breeding programmes seek improvements in areas ranging from fruit quality (e.g. seedlessness, acid content) to resistance against emergent diseases (e.g. huanglongbing). Unfortunately, breeding efforts are hampered by a long juvenility period of 6 or more years, characterized by thorniness, lack of flowering and vertical as opposed to spreading growth form (Spiegel‐Roy and Goldschmidt, 1996).

Genetic transformation of citrus rootstock varieties has produced early flowering. ‘Carrizo’ citrange (*Citrus sinensis* × *Poncirus trifoliata*) constitutively expressing *Arabidopsis thaliana* floral identity genes *LEAFY* (*LFY*) or *APETALA1* (*AP1*) flowered and set fruit 12–20 months after transfer to the greenhouse (Peña *et al*., 2001). In trifoliate orange (*P. trifoliata*), ectopic expression of Satsuma mandarin (*C. unshiu*) *FLOWERING LOCUS T* (*CiFT*) led to flowering as early as 12 weeks after transfer to the greenhouse (Endo *et al*., 2005). However, ectopic expression of flowering genes in citrus has been associated with aberrant phenotypes such as dwarfing, curled leaves and a weeping growth form (Endo *et al*., 2005; Peña *et al*., 2001). In contrast, expression of *FT* using a *Citrus leaf blotch virus* (CLBV) vector inoculated into the non‐commercial *C. excelsa* and hybrids produced as part of a citrus breeding programme resulted in early flowering with minimal effects on growth form (Velázquez *et al*., 2016).

Efforts to develop transgenic edible citrus constitutively expressing floral‐inducing genes have encountered difficulty. Transgenic ‘Duncan’ grapefruit (*Citrus* × *paradisi*) and ‘Hamlin’ sweet orange (*C*. *sinensis*) constitutively expressing *FT* genes produced flower buds in tissue culture and failed to produce shoots or whole plants (Moore *et al*., 2016). The authors hypothesized that reducing FT activity in transgenic citrus using non‐constitutive or inducible promoters might allow regeneration of edible citrus plants with a precocious blooming phenotype.

Translational fusions of FT to GFP produced a milder early flowering phenotype in *A. thaliana* compared to unfused FT (Corbesier *et al*., 2007). This raises the possibility that chimeric FT proteins could be expressed constitutively in transgenic edible citrus to produce plants with a precocious blooming phenotype. Here, we expressed *P. trifoliata FT1* (*PtFT1*) as a translational fusion with a single‐chain variable fragment antibody (scFv; Pack and Plückthun, 1992) that is part of a separate study. A constitutive expression cassette was used that included the *Cauliflower mosaic virus* (CaMV) 35S promoter with a double enhancer region (CaMV 35Sp), the *Tobacco etch virus* 5′ untranslated region and the CaMV 35S polyadenylation signal (35S^t^; Restrepo *et al*., 1990). The *PtFT1‐scFv*‐coding region included the *PtFT1* cDNA sequence, a flexible linker sequence ([gly_4_ser]_4_), the *scFv* sequence and a C‐terminal cMyc epitope tag (Figure 1a).

**Figure 1:**
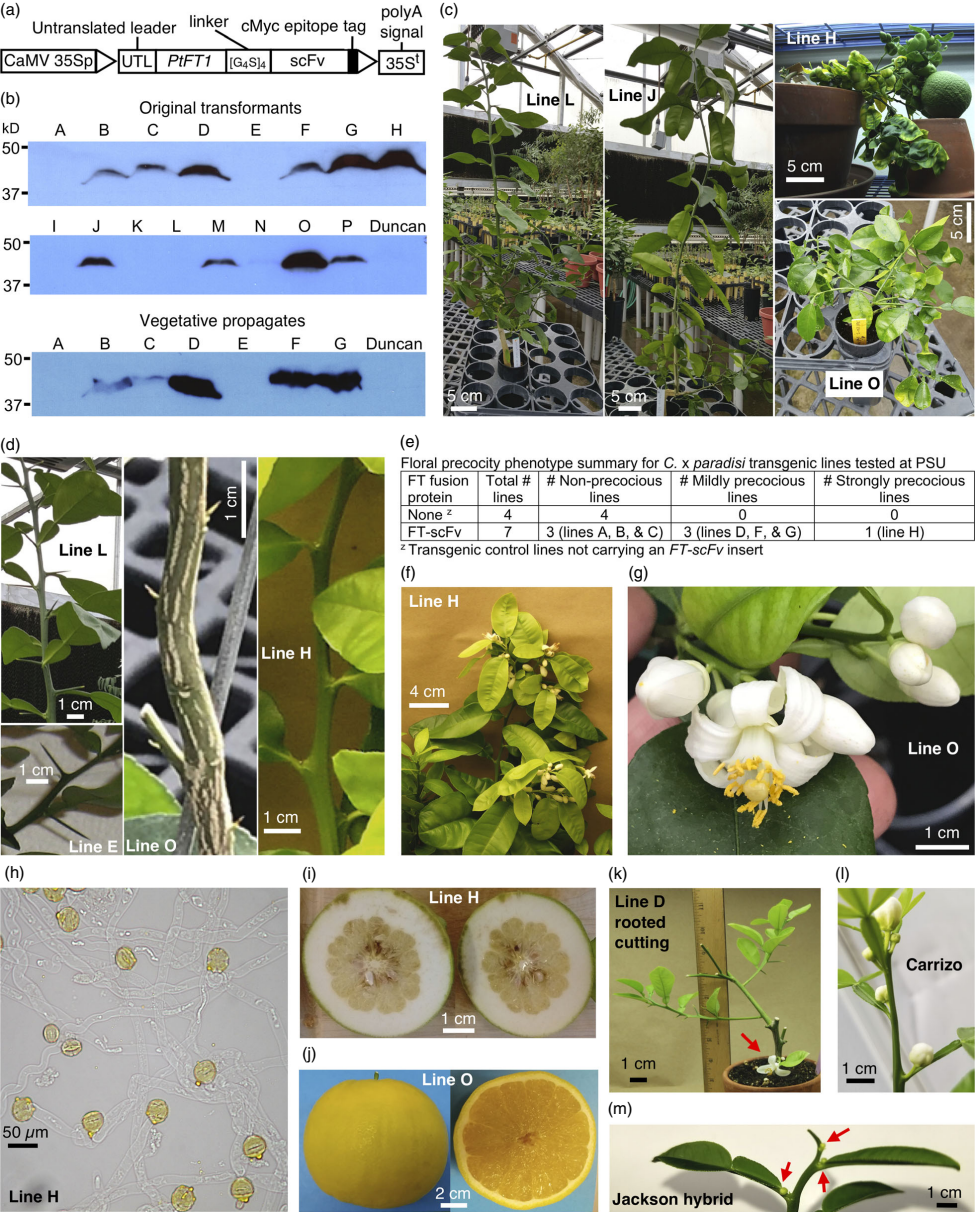
Chimeric PtFT1 fusion protein expression in transgenic grapefruit produces blooming precocity. (a) *PtFT1‐scFv* constitutive expression cassette. (b) Detection of 47.9 kilodalton (kD) PtFT1‐scFv protein in shoot tip extracts using anti‐cMyc immunoblotting; ‘Duncan’, untransformed grapefruit; and protein loading normalized to tissue weight. (c) Growth form of low‐ (line L), moderate‐ (line J) and high‐expressor (lines H and O) plants. (d) Thorniness in transgenic control (line E), low (line L) and high expressors (lines O and H). (e) Phenotype summary. (f) Precocious flowering in a strongly precocious line. (g) Flower morphology in a strongly precocious line. (h) Germinated pollen grains. (i) Seed set in an immature fruit from a hand‐pollinated flower at 5 months after pollination. (j) Ripe fruit from an unpollinated flower. (k) Flower (arrow) on rooted clone of a mildly precocious line. (l,m) Precious phenotypes of ‘Carrizo’ rootstock and ‘Jackson’ hybrid grapefruit *PtFT1‐scFv* transformants. Arrows in (m) indicate flower buds. [Colour figure can be viewed at wileyonlinelibrary.com]

*Agrobacterium tumefaciens* was used to transform ‘Duncan’ grapefruit with the *PtFT1‐scFv* expression construct. Fifteen successful transformants were grafted onto ‘Carrizo’ citrange rootstocks and maintained in a growth chamber at The Pennsylvania State University (PSU; lines A–D, F–H) and a greenhouse at the United States Horticultural Research Laboratory (USHRL; lines I–P). Transgenic control line E, also maintained at PSU, was transformed with a construct lacking *PtFT1‐scFv*.

Full‐length PtFT1‐scFv protein was detected in growth chamber‐ and greenhouse‐grown plants (Figure 1b). PtFT1‐scFv levels varied among the lines, an observation consistent with previous *FT* overexpression studies in trees (Endo *et al*., 2005; Srinivasan *et al*., 2012). PtFT1‐scFv protein was detected in both shoot tips and mature leaves, and was detectable over the course of the four‐year experiment period (data not shown).

Grapefruit trees transformed with *PtFT1‐scFv* displayed varying reductions in juvenile characters that generally correlated with PtFT1‐scFv protein level. For example, line L, which did not have detectable PtFT1‐scFv protein (Figure 1b), largely resembled a juvenile tree, with an upright growth habit and long thorns in the leaf axils (Figure 1c,d). Moderate expressor lines, such as J (Figure 1b), retained an upright growth habit, but had reduced thorn size and occasional moderate leaf curling (Figure 1c). Severe growth phenotypes were observed in the highest expressing lines, H and O, and to a lesser extent in line G. These trees displayed a highly branched, prostrate, dwarfed growth form (Figure 1c) with greatly reduced thorniness or a complete absence of thorns (Figure 1d). Control transgenic line E displayed no reduction in thorniness or other juvenile characters (Figure 1d). Endo *et al*. (2005) similarly reported that higher expression of *CiFT* in transgenic *P. trifoliata* correlated with reduced prevalence of thorns and shorter tree stature.

Precocious flowering was observed in *PtFT1‐scFv* transgenic lines, with the earliest and most frequent flowering being observed in high‐expressing lines H and O. Moderate‐ and high‐expressing lines generally flowered within six months of transfer to soil, with H and O blooming within ~ 3 months. Transgenic lines at PSU were carefully monitored and fell into three blooming phenotype categories: strongly precocious, with large blooming flushes nearly continuously; mildly precocious, blooming 1–4 times a year with isolated flowers; and non‐precocious, which did not bloom during the four‐year monitoring period (Figure 1e). These phenotypic categories generally correlated with FT‐scFv protein levels (Figure 1b) and agreed with previous studies showing that constitutive *FT* expression in trees can break down seasonality of flowering (Endo *et al*., 2005; Srinivasan *et al*., 2012). Transgenic line G had strongly reduced juvenility but did not flower continuously, possibly due to having somewhat lower FT‐scFv levels compared to line H (Figure 1b).

In lines H and O, flushes arose on multiple branches (Figure 1f), usually in leaf axils on leafy inflorescences. Leafless inflorescences were also observed on some shoots. Inflorescences displayed normal development patterns, with the terminal flower usually being the first to reach anthesis (Figure 1f; Spiegel‐Roy and Goldschmidt, 1996).

Flowers produced by *PtFT1‐scFv* trees were fragrant and morphologically normal, with large petals flanking pollen–laden stamens and a central pistil (Figure 1g). Pollen grains were germinated in Brewbaker's medium (Figure 1h; Brewbaker and Kwack, 1963). Hand pollination of flowers with pollen from the same tree resulted in fruit production and seed set (Figure 1i). Fruit development was also observed on several lines without manual pollination, including lines H (Figure 1c) and O, within the first year of transfer to the growth chamber or greenhouse. These fruits ripened normally but were seedless (Figure 1j).

Clonal propagation of low‐ and moderate‐expressing lines via rooted cuttings was highly successful. While line H cuttings never produced roots, rooting was achieved for line O, albeit at a lower rate than moderate and low expressors. Relative PtFT1‐scFv levels in clonal propagates were consistent with those in the original transformants (Figure 1b). Rooted cuttings of *PtFT1‐scFv* transgenic lines maintained the blooming precocity phenotype (Figure 1k).

Our results document the successful use of a chimeric FT protein to reduce flowering time in an edible citrus cultivar. In addition, we developed eight ‘Carrizo’ *FT‐scFv* transformants, of which three had a precocious blooming phenotype (Figure 1l), and one transformant of a hybrid of ‘Jackson’ grapefruit, which had a precocious blooming phenotype (Figure 1m). While very high expression of PtFT1‐scFv was associated with phenotypes that may alter agronomic fitness, moderate expression of the protein resulted in precocious blooming largely without negative effects. FT fusion proteins may have attenuated flowering promotion activity relative to native FT (Corbesier *et al*., 2007), possibly accounting for the success of our approach. The reduced juvenility offered by transgenic expression of chimeric FT proteins may provide an additional valuable tool for rapid‐cycle citrus breeding (Moore *et al*., 2016). Continued studies are underway to evaluate how *C*. × *paradisi PtFT1‐scFv* lines perform in a grove setting.

## Conflict of interest

The authors declare no conflicts of interest.

## Author contributions

T.W.M. designed the study. J.B.H., J.P.S., C.V. and T.W.M. wrote the paper with contributions from all authors. J.P.S., T.W.M., S.M.K., J.B.H., C.V., V.O., E.L.T. and E.S. performed experiments and analysed data. All authors participated in data interpretation for the manuscript.
